# Using Transesophageal Echocardiography in Liver Transplantation with Veno-Venous Bypass Is a Tool with Many Applications: A Case Series from an Italian Transplant Center

**DOI:** 10.3390/jcdd10010032

**Published:** 2023-01-16

**Authors:** Amedeo Bianchini, Cristiana Laici, Martina Bordini, Matteo Bianchin, Catalin Iustin Ioan Silvas, Matteo Cescon, Matteo Ravaioli, Giovanni Vitale, Antonio Siniscalchi

**Affiliations:** 1 Post-Surgical and Transplant Intensive Care Unit, Department of Digestive, Hepatic and Endocrine-Metabolic Diseases, IRCCS Azienda Ospedaliero-Universitaria di Bologna, 40138 Bologna, Italy; 2 Department of Medical and Surgical Sciences (DIMEC), University of Bologna, 40126 Bologna, Italy; 3 Department of Digestive, Hepatic and Endocrine-Metabolic Diseases, IRCCS Azienda Ospedaliero-Universitaria di Bologna, 40138 Bologna, Italy; 4 Hepatobiliary and Transplant Surgery Unit, IRCCS Azienda Ospedaliero-Universitaria di Bologna, 40138 Bologna, Italy; 5 Internal Medicine Unit for the Treatment of Severe Organ Failure, IRCCS Azienda Ospedaliero-Universitaria di Bologna, 40138 Bologna, Italy

**Keywords:** hemodynamic instability, liver transplant, transesophageal echocardiography, veno-venous bypass

## Abstract

Background: Hemodynamic instability (HDI) is common during liver transplantation (LT); veno-venous bypass (VVB) is a tool used in selected cases to ensure hemodynamic stability and for surgical needs. Transesophageal echocardiography (TEE) allows the transplant team to identify the causes of HDI and to guide therapies. We present a case series of four patients showing the valuable role of TEE during LT in VVB. Methods: We report four explicative cases of TEE use in LT with VVB performed at IRCCS Azienda Ospedaliero–Universitaria di Bologna. Four transplants were performed between 2016 and 2022. Results: Many authors have highlighted the diagnostic value of TEE during LT in the case of HDI. However, its specific role during LT with VVB is poorly described. This paper illustrates multiple potential uses of TEE in LT with VVB: TEE as a guide for catheterization and optimal cannula positioning, TEE as a tool for intraoperative Patent Foramen Ovale management, TEE as help for anticoagulation therapy and finally, TEE as support when evaluating bypass efficiency and correcting hypovolemia. Conclusion: TEE is a useful instrument during LT with VVB. However, further studies are needed to assess the suitable applications of TEE during LT in patients with HDI requiring VVB. TEE should be part of the anesthetist’s cultural background.

## 1. Introduction

In the last two decades, the use of intraoperative transesophageal echocardiography (TEE) as a monitoring tool has constantly increased in non-cardiac surgery settings, such as orthotopic liver transplantation (LT) [1]. Intraoperative TEE examination assesses biventricular function, volume status, cardiac output, valvular pathology and systolic anterior motion (SAM) diagnosis [2,3]. Furthermore, TEE is a beneficial tool for the early identification of the causes of intraoperative new-onset hemodynamic instability (HDI). HDI occurs to one degree or another during almost all liver transplants [4]. Multiple factors predispose to intraoperative HDI: the hemodynamic status of the patient with end-stage liver disease, the pre-existing cardiovascular disease and the surgical phases. The main expected hemodynamic changes due to surgery are the acute reduction in preload (total caval clamping and massive bleeding) and the reperfusion syndrome (acute decrease in systemic vascular resistance and/or new-onset ventricular dysfunction). The preload reduction can be mitigated using a partial cava clamp (piggyback technique) or veno-venous bypass (VVB).

VVB consists of extracorporeal circulation from the femoral vein (drainage cannulae) to the internal jugular vein (return cannulae), allowing better hemodynamic stability. An additional cannula for the portal vein (drainage cannulae) also allows venous decompression of the splanchnic circulation with reduced venostasis and intraoperative bleeding and facilitates surgical activity [4]. However, VVB can also cause serious complications, which include bleeding, thrombosis, hemolysis, infections and technical and mechanical problems. The most reported complications (2.2% of the LTs with VVB) are hemomediastinum, air embolism, low flow rate, hypotension, and atrial fibrillation [5].

There is growing evidence that TEE can influence the anesthetic management during LT: numerous clinical conditions such as ventricular dysfunction, valvular regurgitation, air embolization, intracardiac defects and pericardial tamponade have been reported to be detected by intraoperative TEE that would not have been noticed with other monitoring methods, including pulmonary artery catheter (PAC) [6]. TEE assumes a unique additional role in LT with VVB: it allows us to verify the correct positioning of the cannula, to identify the onset of complications and to monitor the hemodynamics during the bypass run [7].

In our center all liver transplants in VVB are monitored with a Swan–Ganz pulmonary catheter. Approximately less than half of these patients are additionally monitored with TEE by the transplant anesthetist. However, not all cases undergo TEE monitoring due to the limited number of adequately trained anesthesiologists, the presence of contraindications (e.g., recent gastrointestinal bleeding) or the temporary unavailability of the transesophageal probe [4].

Herein we present four clinical cases showing how TEE intraoperative monitoring improved the anesthetic management of the patients at risk of HI during LT in VVB.

## 2. Materials and Methods

We retrospectively collected the clinical data of four peculiar cases who underwent LT at IRCCS Azienda Ospedaliero–Universitaria di Bologna, Italy, between January 2016 and November 2022, in whom TEE was used during the surgery and extracorporeal circulation via VVB. A GE LOGIQ 7 ultrasound machine with a GE 6T transesophageal probe was used by a trained anesthetist. In patients in whom TEE was not performed, cannulae were placed under fluoroscopy. This study was conducted in accordance with the ethical guidelines of the World Medical Association’s Declaration of Helsinki and guidelines for Good Clinical Practice [8,9]. Finally, we have attached the more explanatory images of cases 3 and 4; in case 1, there were no good-quality images, and in case 2, it was impossible to photograph the occurrence of pulmonary microemboli.

## 3. Results

### 3.1. Overall Results

**Figure 1:**
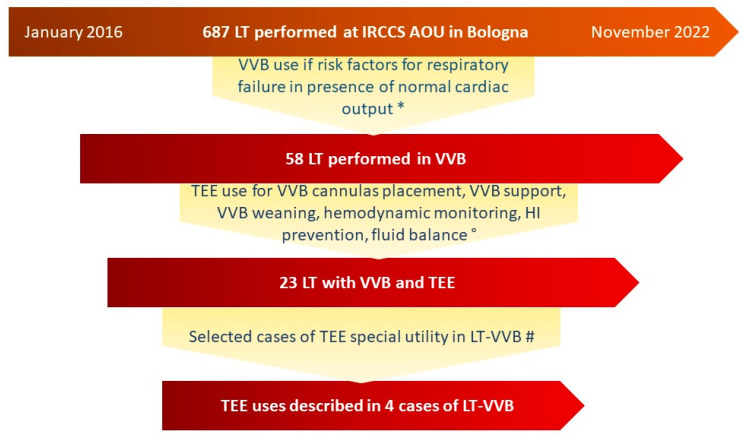
Multidisciplinary Decision Flowchart of TEE and VVB use in patients undergoing LT at IRCCS Azienda Ospedaliero–Universitaria di Bologna. * Risk-benefit balance of VVB use:- Indication to VVB by assessment of factors predisposing to intraoperative HDI, such as the hemodynamic status of the patient with end-stage liver disease and the aetiology of liver failure, pre-existing cardiovascular disease, the complexity of surgical phases and the risk of reperfusion syndrome (acute decrease in systemic vascular resistance or new-onset ventricular dysfunction); Contraindication to VVB by a risk assessment of related complications such as bleeding, arrhythmias, pneumothorax, thrombosis and anaemia. ° Since VVB can cause severe technical complications such as hemomediastinum, air embolism, low flow rate, hypotension and atrial fibrillation, a case-by-case assessment of TEE use was evaluated, also depending on the availability of the transesophageal probe and adequately trained anesthesiologists. # We selected the four cases of TEE use in LT–VVB to underline the unique addition of TEE in this setting: it allows for verification of the correct positioning of the cannula, identifies the onset of complications and monitors the hemodynamics during the bypass run: in case 1, TEE use discovered a new-onset acute pericardial effusion with reduced right ventricle diastolic filling and allowed the execution of emergency sternotomy; in case 2, during the total caval clamping, a pulmonary microembolism was identified thanks to TEE; in case 3, the TEE allowed identification of a moderate-grade PFO. Therefore, the outflow cannula was retracted approximately 10 mm to direct the flow away from the PFO using a colour-Doppler-guided approach with TEE ME-bicaval view. During surgery, we constantly verified the absence of a right-to-left shunt; in case 4, a patient who had previously undergone percutaneous occlusion of PFO, TEE allowed the introduction of the Super Stiff Guidewire from the right internal jugular vein to the inferior vena cava without hitting the atrial occlusion. Using the colour-Doppler guide, we avoided the bypass flow directed towards the occluder. After cannulation, we checked the integrity of the occluder using B-mode ultrasound in the ME-bicaval view and ME-short axis aortic valve view. HI: Hemodynamic Instability; LT: Liver Transplantation; ME: Mid-Esophageal; PFO: Patent Foramen Ovale; TTE: Trans-Esophageal Echocardiography; VVB: Veno-Venous Bypass.

From January 2016 to November 2022, 687 LTs were performed at IRCCS Azienda Ospedaliero–Universitaria of Bologna, Italy. Of these, 58 were conducted with VVB assistance. Twenty-three LT with VVB patients received TEE during the surgical phases. Figure 1 summarizes the Multidisciplinary Decision Flowchart regarding TEE and VVB use in patients undergoing LT in our Transplant Center. We selected four peculiar cases of TEE use in LT–VVB to underline the unique addition of TEE in this setting: it allows for verification of the correct positioning of the cannula, identifies the onset of complications and monitors the hemodynamics during the bypass run. Below we describe the case series in detail.

### 3.2. Case Presentation

#### 3.2.1. Case 1

A 49-year-old female patient with polycystic liver disease and severe abdominal distension was studied for LT in our center in 2016. The preoperative echocardiography showed the absence of heart disease. Due to the surgical complexity with the need to perform total caval clamping, we assisted the patient via a VVB. After the induction of general anesthesia, a right femoral vein (21 Fr cannula) and right internal jugular vein (17 Fr cannula) cannulation were performed. Cannula placement was subsequently verified with a TEE examination that revealed the jugular cannula tip positioned in the upper portion of the right atrium. During surgery, advanced hemodynamic monitoring consisted of PAC and TEE. Throughout the surgery the bypass flow was maintained between 1.5 and 3 L/min with a standard circuit range of pressures. A long and laborious dissection phase with substantial hemodynamic stability characterized the surgery. Following portal reperfusion, we observed a sudden hemodynamic change with hypotension, tachycardia, an increase in central venous pressure (CVP) from 7 mm Hg to 20 mm Hg and desaturation. The HDI onset and PAC data were suggestive of reperfusion syndrome, so fluids and norepinephrine were administered. Subsequently intraoperative TEE showed the presence of a new-onset circumferential pericardial effusion with reduced right ventricle diastolic filling, and marked changes in transmitral blood flow velocities (>25%). An emergency sternotomy was performed, and a right atrial perforation was identified and repaired, obtaining hemodynamic stability and a favourable surgery and patient outcome.

#### 3.2.2. Case 2

A 65-year-old male patient with Hepatitis-C-related cirrhosis complicated by hepatocellular carcinoma and severe portal hypertension was admitted for LT in our center in 2021. Preoperative transthoracic echocardiography (TTE) showed no pathological features. An intraoperative femoro-jugular VVB was required during the surgery for expected surgical complexity: in the history, the patient had performed various locoregional treatments of hepatocellular carcinoma in addition to the placement of a transjugular intrahepatic portosystemic stent-shunt complicated by stent thrombosis. Before starting the extracorporeal circulation, the heparin bolus was not administered due to severe thrombocytopenia (43,000/mm^3^), impaired International Normalized Ratio (INR 2.2) and expected difficult liver dissection. We placed a 21Fr femoral cannula and a 21Fr jugular cannula without complications. VVB flow rate was maintained between 1.5–3 L/min. Hemodynamic monitoring consisted of the Swan–Ganz catheter and TEE, while coagulation assessment was monitored with activated clotting time (ACT) and thromboelastography (TEG). The patient received four concentrated red blood cell units for surgical bleeding during liver dissection. In this phase, during the total caval clamping, a pulmonary microembolism was identified thanks to the visualization of small floating hypoechoic images in the right heart, coming from the outflow cannula and directed towards the pulmonary circulation. The pulmonary microembolism was not causing HDI or hypoxia, and ACT was 215 s. However, a dose of heparin (1000 UI i.v.) was administered to prevent the mobilization of other thrombi during the subsequent graft reperfusion. No further thromboses were visualized and there were no bleeding complications. The post-operative course was regular.

#### 3.2.3. Case 3

A 58-year-old male patient with a history of LT for polycystic liver and kidney disease was scheduled for re-LT for primary graft non-function in 2022. An intraoperative VVB was used for surgical needs. The preoperative TTE showed systemic hypertension-related left ventricular hypertrophy, mitral prolapse and enlargement of the ascending aorta. No alterations of the atrial septum were detected.

The TEE probe was placed after the induction of general anesthesia and tracheal intubation. A moderate-grade Patent Foramen Ovale (PFO) was identified (3 mm). A right-to-left shunt was documented with colour-Doppler and bubble test by applying a positive airway pressure of 20 cm H_2_O for 5 seconds and a positive end-expiratory pressure (PEEP) of 15 cm H_2_O, as described by Koroneos et al. [10].

**Figure 2:**
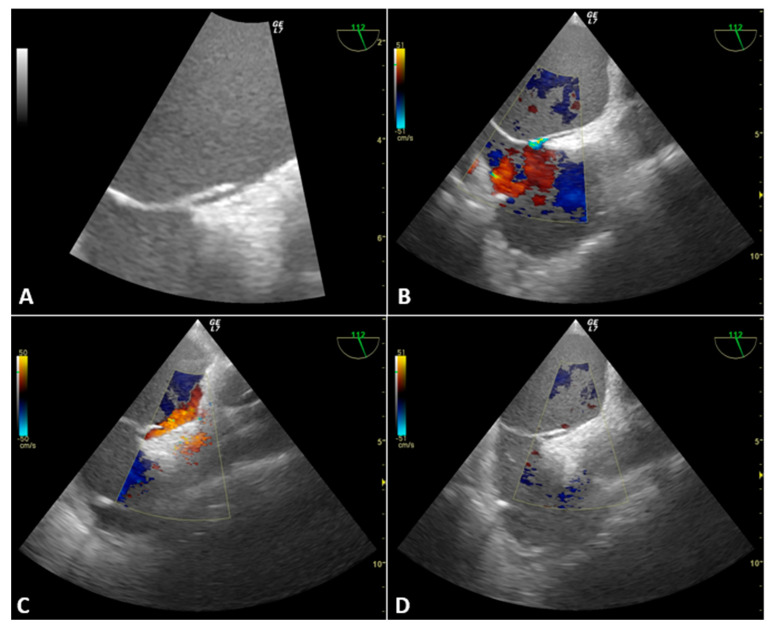
Case 3. (**A**) Patent Foramen Ovale. (**B**) Left-to-right shunt in basal conditions during general anesthesia (**C**) Right-to-left shunt during a positive airway pressure test (20 cm H_2_O) (**D**) Absence of PFO shunt after graft reperfusion despite moderate reperfusion syndrome, bypass outflow positive pressure and mechanical ventilation.

After starting the VVB flow, the outflow cannula (17Fr) was retracted (Figure 2) approximately 10 mm to direct the flow away from the PFO using a colour-Doppler-guided approach with TEE mid-esophageal (ME) bicaval view.

During surgery, PEEP was set between 0 and 5 cm H_2_O and the maximum positive airway pressure was 14 cm H_2_O. Depending on the surgical phase, the bypass flow ranged between 1.5 and 2.5 L/min. Pulmonary and central venous pressure (CVP) increased moderately after graft reperfusion (CVP from 2 to 10 mm Hg). During surgery, the absence of a right-to-left shunt was constantly verified despite the increase in right atrium pressure due to mechanical ventilation, VVB outflow positive pressure and the onset of a moderate reperfusion syndrome. There were no intra- or postoperative cardioembolic complications.

#### 3.2.4. Case 4

**Figure 3:**
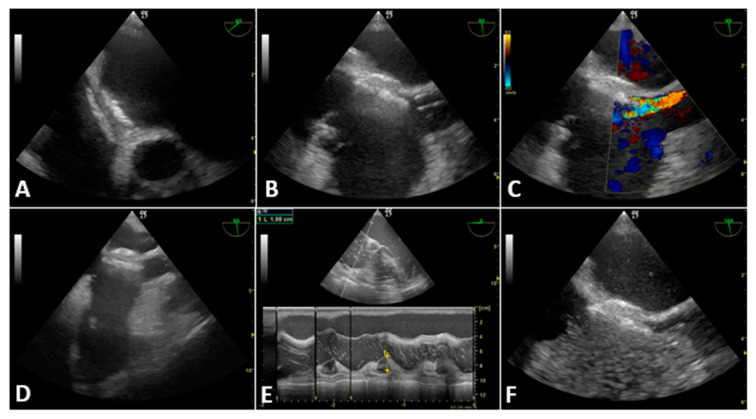
Case 4. (**A**) PFO occluder in mid-esophageal aortic short-axis view. (**B**) Bypass cannula close to the occluder (before retraction) in mid-esophageal bicaval view (**C**) Bypass flow directed towards the occluder before cannula retraction (**D**) Bypass cannula after its retraction (**E**) Spontaneous bubble test after graft reperfusion: absence of PFO shunt (**F**) Bubble test at the end of the surgery: absence of PFO shunt.

A 41-year-old male patient with Alagille syndrome complicated by severe cholestatic liver disease, PFO and retinopathy was scheduled for LT in 2022. At age 35, he underwent percutaneous closure of the PFO (AGA PFO OCCLUDER 25 mm) for evidence of a severe right-to-left shunt during the Valsalva maneuver. Pre-LT echocardiography documented the correct placement of the atrial devices and the absence of residual shunt. The LT was performed with the VVB technique due to the surgical complexity (previous biliary tract surgery). After the induction of general anesthesia and tracheal intubation, the TEE ultrasound probe was positioned in the ME bicaval view. The central venous catheter and the PAC were then introduced using an ultrasound-guided approach. The Seldinger guide and the catheters were constantly visualized in the superior vena cava and the right atrium to avoid injury or detachment of the atrial occluder. The ultrasound-guided approach was later used for the placement of the 17Fr jugular bypass cannula. Thanks to the ultrasound guidance and the passive patient’s head rotation we initially introduced the Super Stiff Guidewire from the right internal jugular vein to the inferior vena cava without hitting the atrial occluder. The 17F jugular cannula was then introduced with ultrasound guidance up to approximately 5 mm from the atrial devices. Next, we retracted the cannula by a further 5 mm using the colour-Doppler guide to avoid the bypass flow being directed towards the occluder. After cannulation, we checked the integrity of the occluder using B-mode ultrasound in the ME-bicaval view and ME-short axis aortic valve view. During the surgery we also repeatedly performed bubble tests with the Valsalva maneuver (positive airway pressure of 20 cm H_2_O for 5 s) to rule out the occurrence of a right-to-left shunt. The shunt was never displayed during the LT, and the devices remained correctly positioned. Figure 3 shows the different TEE-guided procedures in case 4. There were no intra- or postoperative cardioembolic complications.

## 4. Discussion

HDI during LT results from several overlapping pathophysiological conditions due to the patient characteristics, the phases of surgery and intraoperative complications. PAC still represents the gold-standard monitoring tool during LT. It is used for more than 90% of LT patients in the United States [11]. However, a recent position paper of the Society for the Advancement of Transplant Anesthesia (SATA) states that in recent years a greater use of TEE in LT intraoperative monitoring has been observed, despite the absence of randomized clinical trials that confirm its efficacy [12]. For SATA, the increase in TEE use may be due to a better understanding of hemodynamic physiopathology and to the possibility of the identification of pathological conditions otherwise not identifiable with PAC only, such as cardiac tamponade, acute ischemic heart disease, papillary muscle rupture, intracardiac thrombosis, pulmonary embolism, SAM of the mitral valve, etc. [4,13,14] In addition to its usefulness in diagnosing and monitoring hemodynamic pathophysiological conditions during LT, other TEE applications are emerging, including its role in assisting PAC and VVB cannula placement and evaluating other organs such as lungs and hepatic veins [14,15]. For example, Cavayas et al. [16,17] have recently introduced a new approach for the intraoperative study of hypoxia called transesophageal lung ultrasound (TELU), in which different vascular structures recognizable at TEE are used to mark different lung regions. For these reasons most clinicians agree that TEE may be superior to other monitoring methods during LT [1,13]. A retrospective study conducted in 2020 compared three different monitoring approaches during LT (TEE vs. PAC vs. TEE + PAC), demonstrating a better 30-day mortality outcome and reduced Intensive Care Unit length-of-stay outcome for the monitoring approach TEE + PAC, even if a greater incidence of renal insufficiency with CRRT need has emerged [2]. In addition, the American Society of Anesthesiologists, the Society of Cardiovascular Anesthesiologists, the American Society of Echocardiography and the European Association of Echocardiography all individually have recommended the use of TEE monitoring during LT [14]. However, further evidence is needed to make TEE monitoring in LT a standard technique. A recent review suggests that only 38–56% of centers use TEE routinely during LT, the majority using it only in particular circumstances [18]. Reasons against the routine use of TEE may include the lack of anesthesiologist training and concerns about safety. Historically, TEE has been avoided in LT regarding the risk of bleeding from esophageal varices especially during the anhepatic phase. However, recently several studies have demonstrated that TEE may be safely used in patients undergoing LT, even with known upper gastrointestinal varices [19]. Furthermore, in the data analyzed by the SATA consensus, the TEE complication rate appears to be 0.47% versus the 5–10% rate related to PAC use. There needs to be more clarity on the ideal training pathway to perform TEE for LT. However, the last expert panel consensus strongly recommends availability and expertise in TEE monitoring [20]. Given the clinical implications of TEE use, it should become a standard monitoring tool in LT.

The cases we presented involved integrating TEE examination with routine PAC monitoring to enhance patients’ management in LT with VVB. In the literature, TEE is reported as a guiding tool for the bypass cannulation phase. However, this procedure can expose the patient to life-threatening complications such as rupture of large vessels with consequent massive bleeding and atrial perforation resulting in cardiac tamponade [5,21]. To our knowledge, no studies analyze the role of TEE in evaluating dynamic interactions between the patient’s pathophysiology, the surgical phase and the extracorporeal circulation effects. The patient with end-stage liver disease (ESLD) has many pathophysiological conditions that result in HDI including cirrhotic cardiomyopathy, autonomic dysfunction, obstructive coronary artery disease and severe vasodilation from nitric oxide overproduction. The surgical phases also have a decisive intraoperative hemodynamic impact. Some fundamental aspects such as bleeding (medical and surgical), caval clamping, graft reperfusion, the anti-Trendelenburg position and fluidic restriction can cause HDI [4]. VVB improves hemodynamic stability by optimizing venous return to the heart and ventricular filling. TEE can evaluate the filling status of the heart chambers, alert the need to check the bypass flow and guide fluid therapy in the case of hypovolemia. However, VVB can also determine direct or indirect complications that compromise the hemodynamic stability and the patient’s outcome such as cardiac tamponade, pulmonary embolism (both thrombotic and gas emboli) and hemorrhage. In addition, bypass can also potentially facilitate paradoxical embolism through a PFO or atrial defect, especially in the post-graft reperfusion phase, in the case of pulmonary embolism (PE) and patients with porto-pulmonary hypertension (PoPH) [22].

In case 1, the anesthetist had a solid but erroneous suspicion that the hemodynamic changes were due to graft reperfusion syndrome. The TEE examination revealed a completely unexpected diagnosis that changed the patient’s management and prognosis. Atrial perforation is a rare but known complication of central-vein cannulation for VVB [23]. During the liver dissection and venous clamping phase, fluid restriction, low PEEP and the anti-Trendelenburg position limited the pericardial effusion formation. Subsequently, the effusion rapidly increased with the rise in venous return, pulmonary pressure and right atrial pressure due to graft reperfusion. Only TEE allowed the differential diagnosis between cardiac tamponade and reperfusion syndrome. In case 2, we present a patient with a high thrombotic risk (neoplasia, total caval clamping) and factors for increased bleeding risk (multiple blood transfusions, difficult adhesiolysis, hypothermia, liver failure). Anticoagulation targets and coagulation management are still highly debated in LT with VVB. In many centers there is a tendency not to administer anticoagulants due to the high risk of bleeding during complex adhesiolysis or the anahepatic phase (severe spontaneous alteration of coagulation). Indeed, these patients have a known risk of extra- or intra-cardiac thrombosis [24]. Several cases of intracardiac thrombosis (ICT) during LT successfully diagnosed with TEE have been reported in the literature [25,26]. Some data support that without the routine use of TEE during LT, the incidence of ICT will remain an under-recognized event [27]. In our experience, TEE can guide the management of anticoagulant therapy (integrating with ACT and TEG values) thanks to the possibility of visualizing intracardiac microthrombi or thrombotic risk factors such as spontaneous echo contrast. Finally, cases 3 and 4 demonstrate the unique role of TEE in PFO diagnosis and shunt monitoring. PFO has a high prevalence in normal adults (approximately 25% of individuals), decreasing with age [28]. It is usually associated with a small amount of left-to-right shunt and asymptomatic. However, PFO is associated with a significant increase in the odds of stroke in non-cardiac surgery settings [29]. An increase in right atrial pressure (such as in the Valsalva maneuver) favours the reversal of the right-to-left shunt. After graft reperfusion, PE or PoPH, there is a rise in right atrial pressure with a possible shunt reversal and a high risk of paradoxical embolism. TEE allows the constant evaluation of the onset of the right-to-left shunt. Thus, it could help guide therapies to reduce atrial pressure (e.g., diuretic therapy, ventilatory pressure reduction, use of nitric oxide and inodilators). Furthermore, TEE favours placing the bypass cannula to direct the outflow away from the PFO and reduce the onset of paradoxical embolism. Hemamalini et al. suggested that TEE is the ideal modality to guide cannula placement compared to fluoroscopy [30]. This case series finally shows how TEE can change and improve intraoperative anesthetic management during LT, especially in VVB. The absence of TEE monitoring would probably have led to cardiorespiratory arrest in case 1 and pulmonary or systemic thromboembolic complications (paradoxical thrombi) in the other patients. Furthermore, traumatic complications could occur in all patients (e.g., atrial perforation), and the atrial occluder could be damaged or dislocated in patient 4.

This result stimulates the routine use of TEE during LT with VVB (if not contraindicated) and, consequently, the adequate training of the entire anesthesiology team.

## 5. Conclusions

Evidence of monitoring strategies’ superiority with PAC and TEE during LT is growing. TEE allows us to quickly identify the causes of HDI and guide the therapies to support volume status, biventricular function and vascular resistances. It has also been helpful in particular situations, such as LT with VVB. It can guide patient management and be a diagnostic tool in these circumstances. However, further studies are needed. TEE knowledge and practice should be part of the anesthesiologist’s cultural background.

## Data Availability

Data supporting reported results can be found at IRCCS Azienda Ospedaliero–Universitaria di Bologna, department of digestive, hepatic and endocrine-metabolic diseases. Post-Surgical and Transplant Intensive Care Unit.

## Abbreviations

ACT	Activated Clotting Time
CVP	Central Venous Pressure
ESLD	End-Stage Liver Disease
Fr	French
HDI	Hemodynamic Instability
ICT	Intra-Cardiac Thrombosis
LT	Liver Transplantation
ME	Mid-Esophageal
PAC	Pulmonary Artery Catheter
PE	Pulmonary Embolism
PEEP	Positive End-Expiratory Pressure
PFO	Patent Foramen Ovale
PoPH	Porto-Pulmonary Hypertension
SAM	Systolic Anterior Motion
SATA	Society for the Advancement of Transplant Anesthesia
TEE	Trans-Esophageal Echocardiography
TEG	Thrombo-Elasto-Graphy
TELU	Trans Esophageal Lung Ultrasound
TTE	Trans-Thoracic Echocardiography
VVB	Veno-Venous Bypass
